# A Case of Concurrent Tricuspid Valve, Mitral Valve, and Device Endocarditis

**DOI:** 10.7759/cureus.41654

**Published:** 2023-07-10

**Authors:** Fahad Hussain, Carmel Moazez, Revati Reddy, Jerome Yatskowitz, Mark Garcia, Alex Schevchuck

**Affiliations:** 1 Internal Medicine, Northwell Health, Manhasset, USA; 2 Internal Medicine, University of New Mexico, Albuquerque, USA; 3 Cardiology, University of New Mexico, Albuquerque, USA

**Keywords:** echocardiography, ventricular septal defect, device endocarditis, mitral valve endocarditis, tricuspid valve endocarditis

## Abstract

Endocarditis involving multiple valves is a relatively rare phenomenon, and much about its etiology, prognosis, and best practices for treatment remains uncharacterized. Currently, the treatment of multiple-valve endocarditis is similar to that of single-valve endocarditis. However, limited data suggest that patients may potentially benefit from different treatment approaches not yet clearly defined. Here, we present a unique case of a 22-year-old female with a history of aortic coarctation repair and a ventricular septal defect (VSD) patch repair who presented to the emergency department (ED) after acute onset of fever, chills, nausea, vomiting, and diarrhea. The patient was admitted to the ICU with septic shock and was found to have concurrent mitral valve, tricuspid valve, and VSD patch endocarditis. We discussed her hospital course and treatment as well as current treatment approaches to multiple-valve endocarditis.

## Introduction

Infective endocarditis is an infection of the endocardial surface of the heart, typically involving one or more valves or an intracardiac device [1]. Although the exact prevalence in the population is unknown, endocarditis involving multiple valves is rare. A prospective and observational study conducted across three tertiary centers involving 680 cases of endocarditis found only 17% to exhibit multiple valve involvement [2]. This same study found that although multiple-valve endocarditis was more frequently associated with complications such as heart failure and perivalvular disease, in-hospital mortality was similar to single-valve endocarditis. Additionally, the most frequent combinations described in the study were native mitral valve-native aortic valve (46%), mechanical mitral valve prosthesis-mechanical aortic valve prosthesis (14%), and native mitral valve-mechanical aortic valve (9%). Of the small number of multiple-valve endocarditis cases, most tend to involve the aortic and mitral valves.

The concurrence of mitral valve and tricuspid valve endocarditis is exceedingly rare, and the prevalence is unknown. The literature describes cases of concurrent mitral and tricuspid valve endocarditis commonly associated with a ventricular septal defect (VSD) [3,4]. In general, the presence of a VSD, especially if unrepaired, increases the risk of endocarditis [5]. VSD repair is typically done by direct patch closure under cardiopulmonary bypass. Repair is indicated in patients with persistent symptoms despite maximal medical therapy, moderate or large defects associated with pulmonary hypertension, persistent left-to-right shunt with associated left ventricular dilation in the absence of symptoms, sub-pulmonic and membranous defects with associated aortic valve prolapse and aortic regurgitation, or double-chambered right ventricle [6,7]. Although VSD patch repair is associated with lower rates of infective endocarditis compared to unrepaired VSD, there are multiple reported cases of endocarditis involving VSD patches, especially synthetic ones [8,9]. While the literature describes different combinations of valves and devices associated with infective endocarditis, our team could not identify any cases of VSD patch endocarditis in addition to multivalvular involvement. We discuss a unique case of concurrent tricuspid, mitral, and device endocarditis, as well as a treatment approach.

## Case presentation

A 22-year-old female with a history of aortic coarctation repair and a VSD patch repair in infancy presented to the emergency department (ED) after acute onset of fever, chills, nausea, vomiting, and diarrhea. She denied any sick contacts but reported finishing a course of antibiotics for a toe infection a week prior. Additionally, she was treated for pneumonia one month before the presentation. She was ultimately admitted to the intensive care unit with septic shock requiring vasopressors and found to have methicillin-sensitive Staphylococcus aureus (MSSA) bacteremia along with Clostridium difficile colitis. The patient was started on broad-spectrum antibiotics as well as oral vancomycin. She tested negative for COVID-19 at the time of admission.

A transthoracic echocardiogram (TTE) was done on day 5 of her hospital stay to evaluate for suspected endocarditis. The study showed nonspecific abnormalities of mitral and tricuspid valves. The patient was then transferred to the University Hospital for further management and a transesophageal echocardiogram (TEE). She was evaluated for potential sources of infection with a mandible panorex, showing scattered dental caries, and a chest X-ray concerning atypical pneumonia. 

**Figure 1:**
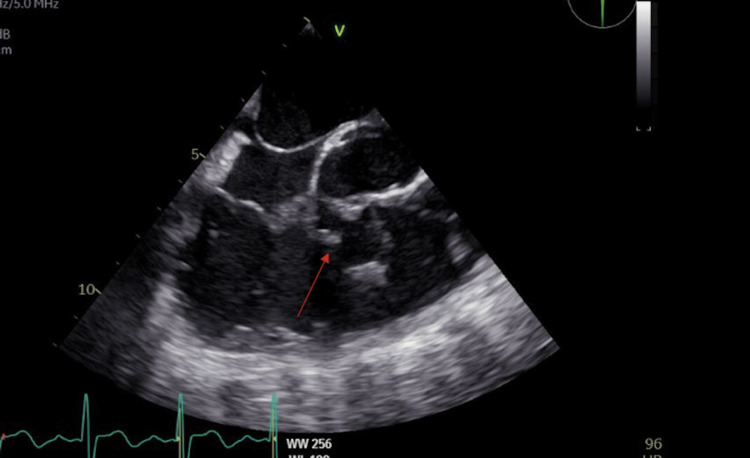
TEE four-chamber view of the VSD patch. TEE, transesophageal echocardiogram; VSD, ventricular septal defect

**Figure 2:**
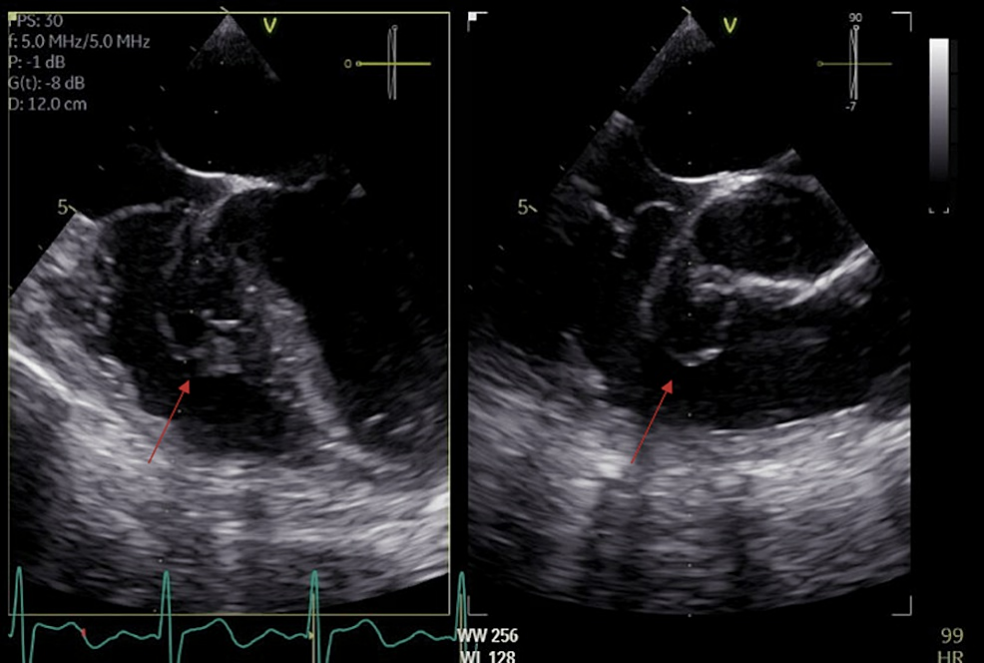
TEE images of the VSD patch in the four-chamber view with anterior and posterior views. TEE, transesophageal echocardiogram; VSD, ventricular septal defect

**Figure 3:**
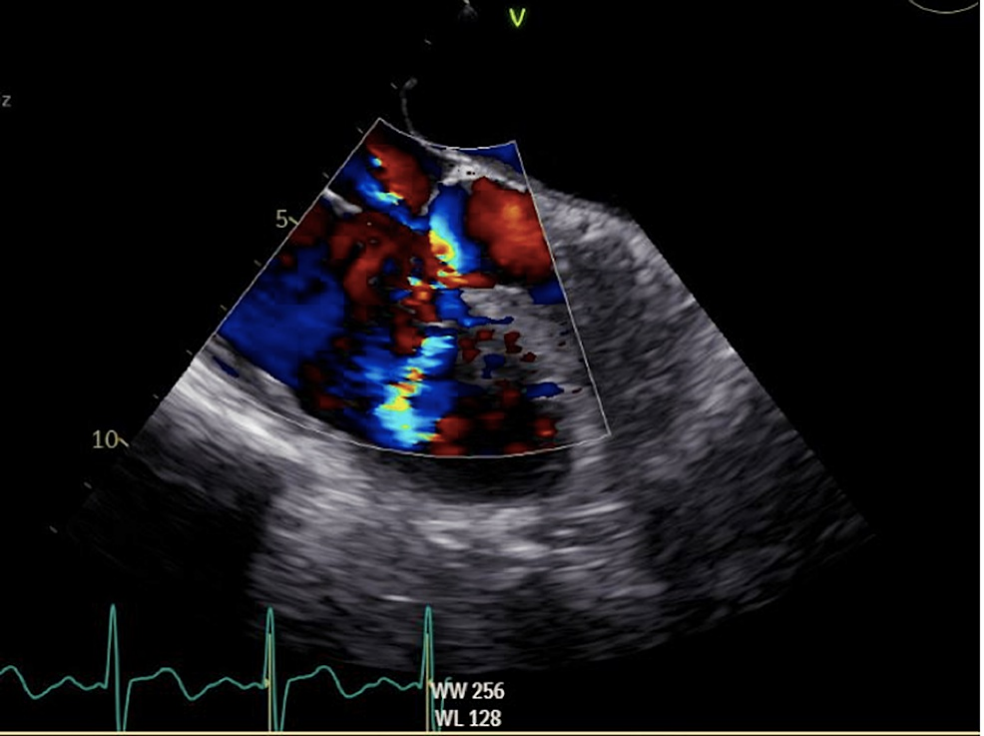
TEE image with color Doppler flow through the VSD. TEE, transesophageal echocardiogram; VSD, ventricular septal defect

**Figure 4:**
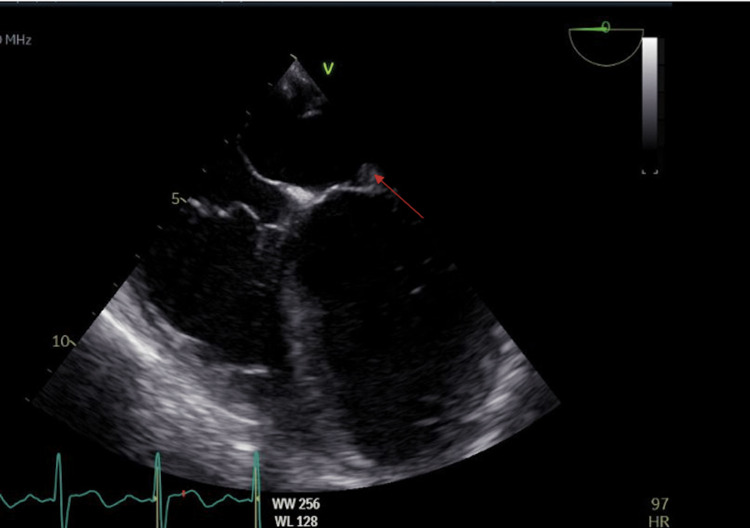
TEE image of the mitral valve vegetation in the four-chamber view. TEE, transesophageal echocardiogram

**Figure 5:**
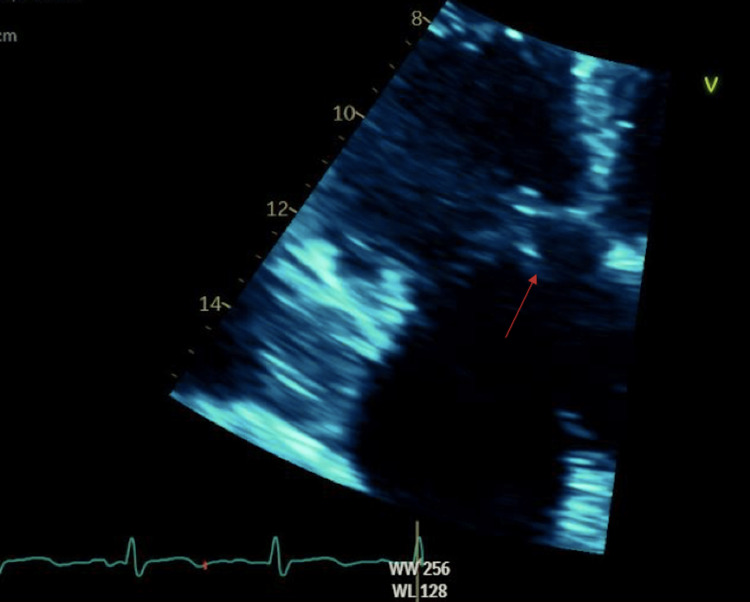
TTE image of the tricuspid vegetation. TTE, transthoracic echocardiogram

TEE was significant for a membranous VSD, and color flow Doppler depicted a VSD with left-to-right shunt along with redundant and mobile tissue in the right ventricle, consistent with dislodgement and endocarditis of the VSD patch and involvement of the tricuspid valve leaflet. Additionally, the posterior annulus of her mitral valve was found to have a small mass consistent with vegetation, mild mitral regurgitation, and a normal ejection fraction (Figures 1-4). A cardiac magnetic resonance imaging (MRI) was significant for mild tricuspid and mitral valve thickening with trace regurgitation of both valves, prior membranous VSD patch repair with Qp:Qs ratio of 1.9, VSD measuring 20 mm with patch bulging into the right ventricle, and the shunt-related jet across the VSD measuring up to 7 mm in width. The findings were suggestive of concurrent tricuspid, mitral, and device endocarditis. A follow-up TTE four days after TEE displayed a small vegetation on the anterior leaflet of the tricuspid valve with mild tricuspid regurgitation, mildly dilated ventricles and left atrium, and normal systolic function of both ventricles (Figure 5). The VSD patch appeared similarly dislodged, as seen on the TEE.

The patient was initially treated for bacteremia. After MSSA specification and imaging diagnosis of endocarditis, therapy was de-escalated to intravenous (IV) nafcillin. Seven days after the initial presentation, blood cultures became clear. C. difficile colitis was treated with a 10-day course of oral vancomycin. Cardiothoracic surgery was consulted and decided to defer surgical intervention until after antibiotic therapy was completed. A peripherally inserted central catheter (PICC) line was placed 72 hours after her negative blood culture. She was ultimately discharged with eight weeks of IV nafcillin followed by long-term suppressive antibiotic therapy with dicloxacillin. The patient was referred for surgical repair of the VSD after completion of the antibiotics course.

## Discussion

Endocarditis involving multiple valves is underrepresented in the literature, and much about its etiology, prognosis, and best practices for treatment are unknown. Currently, multiple-valve endocarditis is treated similarly to single-valve endocarditis, with four to six weeks of antibiotic therapy targeted to the organism isolated from blood cultures, starting after the first negative blood culture result [10]. Valve surgery, performed before completion of antimicrobial therapy, is only considered in some instances displaying complications that are associated with poor prognosis. For left-sided native valve endocarditis, the indications for early surgery include valve dysfunction causing symptoms of heart failure, a perivalvular extension of infection, infection with a multi-drug-resistant organism or fungi, persistent infection, and large vegetation [11]. Indications for early surgery in right-sided native valve endocarditis are similar and include large vegetations, recurrent septic pulmonary emboli, highly resistant organisms, or persistent bacteremia [11]. VSD patch endocarditis is rare, and aside from isolated case reports, there is very little guidance in the literature regarding treatment. In general, most cardiac device-related infections are treated with the removal of the device, followed by variable lengths of treatment with antibiotics and implantation of a new device [12]. Approaches to VSD patch endocarditis described in individual cases typically include the removal of the infected patch and replacement with another patch of similar or different material [9,12].

The largest ongoing study evaluating outcomes in multiple-valve endocarditis found a similar rate of in-hospital mortality in comparison to single-valve endocarditis. However, the study also reported that patients with multiple-valve endocarditis more frequently exhibited complications such as heart failure and perivalvular disease [2]. This study hypothesized that multiple-valve endocarditis is of a similar microbiologic profile to single-valve endocarditis but might represent a more advanced disease [2]. As such, it is possible that patients with multiple-valve endocarditis might benefit from more aggressive treatment or different approaches to treatment than are currently defined.

This patient had mitral and tricuspid valve endocarditis along with involvement and dislodgement of the VSD patch. Most presentations of multiple-valve endocarditis involve the aortic and mitral valves. Concurrent mitral-tricuspid valve endocarditis is much less common. As individual cases involving valves of both the right and left heart have been associated with VSDs, it is possible that the patent VSD in this patient predisposed her to endocarditis of both the mitral and tricuspid valves [3,4]. We hypothesized that the endocarditis initially involved the VSD patch. The patch eventually dislodged, resulting in an open VSD and predisposing to seeding of the two valves, adjacent to the VSD. The patient’s concurrent VSD patch endocarditis, mitral valve endocarditis, and tricuspid valve endocarditis represent a unique case not found in the literature.

## Conclusions

Although much is unknown about multiple-valve endocarditis in conjunction with device endocarditis, there is evidence suggesting that several different treatment approaches may be beneficial. It is possible that patients, such as the one described in the case, might benefit from more aggressive treatment or different approaches to treatment than are currently defined. By highlighting this case, we hope to add to the growing body of literature regarding multiple-valve endocarditis and device endocarditis.
